# Poly[(3,5-dinitro­benzoato)-μ_3_-triazolato-cobalt(II)]

**DOI:** 10.1107/S160053680804155X

**Published:** 2008-12-13

**Authors:** Xu-Liang Qi

**Affiliations:** a Liaocheng Vocational and Technical College, Liaocheng, Shandong, People’s Republic of China

## Abstract

The title compound, [Co(C_2_H_2_N_3_)(C_7_H_3_N_2_O_6_)]_*n*_, was obtained by the reaction of CoCl_2_, triazole and 3,5-dinitro­benzoic acid in a 1:1:1 ratio. The Co centre is in a distorted tetrahedral coordination by three N atoms of three different triazole ligands and one O atom of the 3,5-dinitrobenzoate anion.

## Related literature

For background, see: Park *et al.* (2006 ▶).
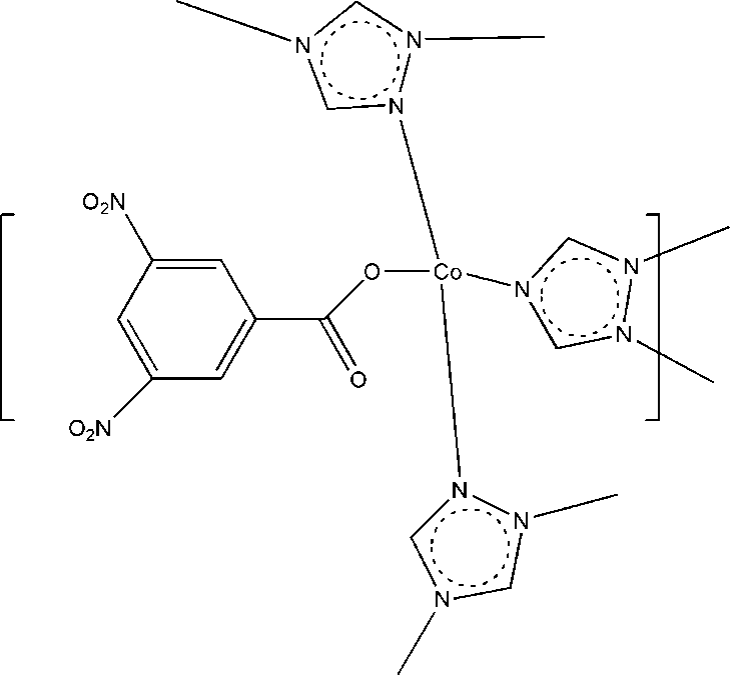


## Experimental

### 

#### Crystal data

[Co(C_2_H_2_N_3_)(C_7_H_3_N_2_O_6_)]
*M* *_r_* = 338.11
Monoclinic, 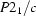

*a* = 11.326 (2) Å
*b* = 9.4043 (19) Å
*c* = 10.696 (2) Å
β = 91.22 (3)°
*V* = 1139.0 (4) Å^3^
*Z* = 4
Mo *K*α radiation
μ = 1.55 mm^−1^
*T* = 296 (2) K
0.14 × 0.12 × 0.10 mm

#### Data collection

Bruker SMART 1K CCD area-detector diffractometer
Absorption correction: multi-scan (*SADABS*; Sheldrick, 2004 ▶) *T*_min_ = 0.812, *T*_max_ = 0.861
10929 measured reflections
2602 independent reflections
2256 reflections with *I* > 2σ(*I*)
*R*_int_ = 0.041

#### Refinement

*R*[*F* ^2^ > 2σ(*F* ^2^)] = 0.026
*wR*(*F* ^2^) = 0.060
*S* = 1.03
2602 reflections
190 parameters
H-atom parameters constrained
Δρ_max_ = 0.33 e Å^−3^
Δρ_min_ = −0.36 e Å^−3^

### 

Data collection: *SMART* (Bruker, 2001 ▶); cell refinement: *SAINT* (Bruker, 2001 ▶); data reduction: *SAINT*; program(s) used to solve structure: *SHELXS97* (Sheldrick, 2008 ▶); program(s) used to refine structure: *SHELXL97* (Sheldrick, 2008 ▶); molecular graphics: *SHELXTL* (Sheldrick, 2008 ▶); software used to prepare material for publication: *SHELXL97*.

## Supplementary Material

Crystal structure: contains datablocks I, global. DOI: 10.1107/S160053680804155X/bt2826sup1.cif

Structure factors: contains datablocks I. DOI: 10.1107/S160053680804155X/bt2826Isup2.hkl

Additional supplementary materials:  crystallographic information; 3D view; checkCIF report

## Figures and Tables

**Fig. 1.**
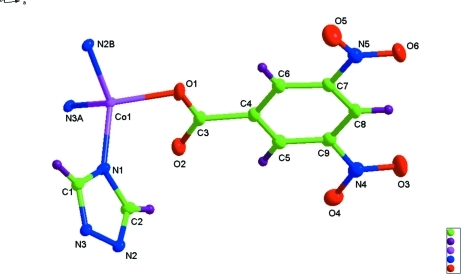
An ORTEP view of the asymmetric unit with 50% displacement ellipsoids for non-H atoms. Symmetry codes: (A) -x,y - 1/2,-z + 1/2; (B) x,-y + 1/2,z + 1/2.

**Fig. 2.**
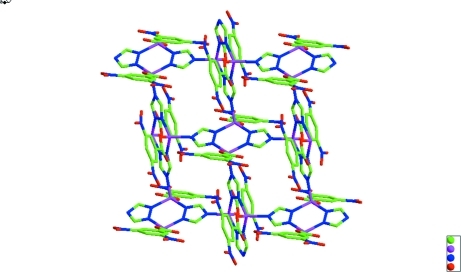
View of the two-dimensional net.

## supplementary crystallographic information

### Comment

The asymmetric unit of the title compound is shown in Fig. 1. Co  is four-coordinated by one O atom of a 3,5-dinitrobenzoic acid anion and three triazole N atoms in a tetrahedral geometry. The Co—O/N bond lengths of 1.9510 (13)–2.0396 (16)Å are in the normal range. The triazole and 3,5-dinitrobenzoic acid ligands adopt tridentate and monodentate coordinating modes, respectively. As shown in Figs. 2a and 2b, cobalt ions are connected by triazole ligands to generate a two-dimensional net with the 3,5-dinitrobenzoic acid ligands stacking out of this net. There is not obvious supramolecular interaction  between the two-dimensional nets.

### Experimental

CoCl_2_ (1.0 mmol), 3,5-dinitrobenzoic acid (1 mmol), and triazole (1 mmol) were dissolved in water (10 ml). The solution was heated in a 25 ml Teflon lined reaction vessel at 433 K for *ca* 3 days and then cooled to room temperature. Purple crystals were obtained in a yield of 85%.

### Refinement

All H atoms were positioned geometrically and refined using a riding model with C—H = 0.93–0.96 Å and with *U*_iso_(H) = 1.2*U*_eq_(C).

### Crystal data

**Table:**

[Co(C_2_H_2_N_3_)(C_7_H_3_N_2_O_6_)]	*F*(000) = 676
*M**_r_* = 338.11	*D*_x_ = 1.972 Mg m^−^^3^
Monoclinic, *P*2_1_/*c*	Mo *K*α radiation, λ = 0.71073 Å
*a* = 11.326 (2) Å	Cell parameters from 8661 reflections
*b* = 9.4043 (19) Å	θ = 3.4–27.5°
*c* = 10.696 (2) Å	µ = 1.55 mm^−^^1^
β = 91.22 (3)°	*T* = 296 K
*V* = 1139.0 (4) Å^3^	Block, red
*Z* = 4	0.14 × 0.12 × 0.10 mm

### Data collection

**Table:**

Bruker SMART 1K CCD area-detector diffractometer	2602 independent reflections
Radiation source: rotating anode	2256 reflections with *I* > 2σ(*I*)
graphite	*R*_int_ = 0.041
ω scans	θ_max_ = 27.5°, θ_min_ = 3.4°
Absorption correction: multi-scan (*SADABS*; Sheldrick, 2004)	*h* = −14→14
*T*_min_ = 0.812, *T*_max_ = 0.861	*k* = −12→12
10929 measured reflections	*l* = −13→13

### Refinement

**Table:**

Refinement on *F*^2^	Primary atom site location: structure-invariant direct methods
Least-squares matrix: full	Secondary atom site location: difference Fourier map
*R*[*F*^2^ > 2σ(*F*^2^)] = 0.026	Hydrogen site location: inferred from neighbouring sites
*wR*(*F*^2^) = 0.060	H-atom parameters constrained
*S* = 1.03	*w* = 1/[σ^2^(*F*_o_^2^) + (0.0193*P*)^2^ + 0.8847*P*] where *P* = (*F*_o_^2^ + 2*F*_c_^2^)/3
2602 reflections	(Δ/σ)_max_ = 0.001
190 parameters	Δρ_max_ = 0.33 e Å^−^^3^
0 restraints	Δρ_min_ = −0.36 e Å^−^^3^

### Fractional atomic coordinates and isotropic or equivalent isotropic displacement parameters (Å^2^)

**Table:**

	*x*	*y*	*z*	*U*_iso_*/*U*_eq_	
C1	−0.01347 (15)	0.35028 (19)	0.25548 (18)	0.0159 (4)	
H1	−0.0595	0.3768	0.3226	0.019*	
C2	0.10642 (16)	0.24145 (19)	0.14215 (18)	0.0176 (4)	
H2	0.1617	0.1761	0.1145	0.021*	
C3	0.32087 (15)	0.02322 (19)	0.30024 (19)	0.0187 (4)	
C4	0.45442 (15)	0.03032 (19)	0.29392 (19)	0.0174 (4)	
C5	0.51022 (16)	−0.0334 (2)	0.19406 (19)	0.0186 (4)	
H5	0.4668	−0.0830	0.1335	0.022*	
C6	0.52033 (16)	0.1050 (2)	0.38296 (19)	0.0181 (4)	
H6	0.4838	0.1486	0.4499	0.022*	
C7	0.64177 (16)	0.1134 (2)	0.37020 (19)	0.0185 (4)	
C8	0.70035 (16)	0.0510 (2)	0.27338 (19)	0.0199 (4)	
H8	0.7819	0.0574	0.2668	0.024*	
C9	0.63179 (16)	−0.0217 (2)	0.18643 (19)	0.0195 (4)	
Co1	0.10473 (2)	0.09672 (2)	0.39456 (2)	0.01158 (8)	
N1	0.05927 (13)	0.23721 (15)	0.25698 (14)	0.0148 (3)	
N2	0.06612 (12)	0.34762 (16)	0.07340 (14)	0.0142 (3)	
N3	−0.01218 (12)	0.41947 (15)	0.14790 (14)	0.0140 (3)	
N4	0.69050 (14)	−0.08206 (18)	0.07799 (17)	0.0236 (4)	
N5	0.71165 (14)	0.19524 (18)	0.46234 (16)	0.0217 (4)	
O1	0.27667 (11)	0.08348 (14)	0.39657 (14)	0.0209 (3)	
O2	0.26354 (12)	−0.03470 (16)	0.21590 (14)	0.0270 (3)	
O3	0.79840 (13)	−0.0720 (2)	0.07570 (16)	0.0399 (4)	
O4	0.63013 (13)	−0.13889 (16)	−0.00391 (14)	0.0277 (3)	
O5	0.65828 (13)	0.25969 (17)	0.54211 (16)	0.0339 (4)	
O6	0.81961 (12)	0.19449 (16)	0.45430 (15)	0.0296 (3)	

### Atomic displacement parameters (Å^2^)

**Table:**

	*U*^11^	*U*^22^	*U*^33^	*U*^12^	*U*^13^	*U*^23^
C1	0.0157 (8)	0.0187 (8)	0.0137 (9)	0.0024 (7)	0.0040 (7)	0.0001 (7)
C2	0.0198 (9)	0.0189 (9)	0.0144 (9)	0.0055 (7)	0.0045 (7)	0.0008 (7)
C3	0.0146 (8)	0.0190 (9)	0.0226 (10)	0.0003 (7)	0.0033 (7)	0.0074 (8)
C4	0.0144 (8)	0.0176 (8)	0.0202 (10)	0.0012 (7)	0.0023 (7)	0.0035 (8)
C5	0.0176 (8)	0.0192 (9)	0.0190 (10)	0.0008 (7)	0.0001 (7)	0.0011 (8)
C6	0.0161 (8)	0.0188 (9)	0.0194 (10)	0.0029 (7)	0.0027 (7)	0.0019 (8)
C7	0.0155 (8)	0.0205 (9)	0.0194 (10)	0.0001 (7)	−0.0015 (7)	0.0034 (8)
C8	0.0135 (8)	0.0246 (9)	0.0217 (10)	0.0032 (8)	0.0020 (7)	0.0050 (8)
C9	0.0188 (9)	0.0214 (9)	0.0184 (10)	0.0058 (8)	0.0044 (7)	0.0024 (8)
Co1	0.01125 (12)	0.01384 (12)	0.00973 (13)	−0.00079 (9)	0.00236 (8)	0.00031 (9)
N1	0.0168 (7)	0.0157 (7)	0.0120 (8)	0.0016 (6)	0.0038 (6)	0.0011 (6)
N2	0.0142 (7)	0.0166 (7)	0.0120 (8)	0.0020 (6)	0.0036 (6)	−0.0009 (6)
N3	0.0129 (7)	0.0174 (7)	0.0117 (8)	0.0025 (6)	0.0036 (6)	−0.0008 (6)
N4	0.0217 (8)	0.0280 (9)	0.0211 (9)	0.0074 (7)	0.0047 (7)	0.0026 (7)
N5	0.0196 (8)	0.0230 (8)	0.0225 (9)	−0.0005 (7)	−0.0021 (7)	0.0034 (7)
O1	0.0128 (6)	0.0242 (7)	0.0260 (8)	0.0001 (5)	0.0055 (5)	−0.0001 (6)
O2	0.0178 (6)	0.0368 (8)	0.0265 (8)	−0.0064 (6)	−0.0004 (6)	0.0003 (7)
O3	0.0182 (7)	0.0697 (12)	0.0321 (10)	0.0115 (8)	0.0057 (6)	−0.0112 (9)
O4	0.0324 (8)	0.0299 (7)	0.0210 (8)	0.0010 (6)	0.0032 (6)	−0.0035 (6)
O5	0.0308 (8)	0.0380 (9)	0.0329 (9)	0.0016 (7)	−0.0004 (7)	−0.0153 (7)
O6	0.0157 (6)	0.0387 (8)	0.0343 (9)	−0.0033 (6)	−0.0041 (6)	0.0045 (7)

### Geometric parameters (Å, °)

**Table:**

C1—N3	1.322 (2)	C7—N5	1.469 (3)
C1—N1	1.345 (2)	C8—C9	1.380 (3)
C1—H1	0.9300	C8—H8	0.9300
C2—N2	1.316 (2)	C9—N4	1.464 (3)
C2—N1	1.350 (2)	Co1—O1	1.9510 (13)
C2—H2	0.9300	Co1—N3^i^	2.0158 (15)
C3—O2	1.228 (2)	Co1—N1	2.0356 (16)
C3—O1	1.287 (2)	Co1—N2^ii^	2.0396 (16)
C3—C4	1.517 (2)	N2—N3	1.381 (2)
C4—C6	1.388 (3)	N2—Co1^iii^	2.0396 (16)
C4—C5	1.389 (3)	N3—Co1^iv^	2.0158 (15)
C5—C9	1.385 (3)	N4—O4	1.223 (2)
C5—H5	0.9300	N4—O3	1.227 (2)
C6—C7	1.387 (3)	N5—O5	1.218 (2)
C6—H6	0.9300	N5—O6	1.228 (2)
C7—C8	1.373 (3)		
			
N3—C1—N1	112.41 (16)	C8—C9—N4	117.87 (16)
N3—C1—H1	123.8	C5—C9—N4	118.99 (18)
N1—C1—H1	123.8	O1—Co1—N3^i^	117.64 (6)
N2—C2—N1	113.05 (16)	O1—Co1—N1	106.65 (6)
N2—C2—H2	123.5	N3^i^—Co1—N1	104.57 (6)
N1—C2—H2	123.5	O1—Co1—N2^ii^	103.88 (7)
O2—C3—O1	125.11 (17)	N3^i^—Co1—N2^ii^	107.62 (6)
O2—C3—C4	119.94 (18)	N1—Co1—N2^ii^	117.09 (6)
O1—C3—C4	114.94 (17)	C1—N1—C2	102.70 (15)
C6—C4—C5	119.94 (17)	C1—N1—Co1	131.85 (13)
C6—C4—C3	120.92 (17)	C2—N1—Co1	125.30 (12)
C5—C4—C3	119.07 (17)	C2—N2—N3	105.55 (15)
C9—C5—C4	118.70 (18)	C2—N2—Co1^iii^	129.80 (12)
C9—C5—H5	120.7	N3—N2—Co1^iii^	124.66 (11)
C4—C5—H5	120.7	C1—N3—N2	106.29 (14)
C7—C6—C4	118.76 (18)	C1—N3—Co1^iv^	125.99 (12)
C7—C6—H6	120.6	N2—N3—Co1^iv^	127.71 (12)
C4—C6—H6	120.6	O4—N4—O3	124.17 (18)
C8—C7—C6	123.08 (18)	O4—N4—C9	118.73 (16)
C8—C7—N5	117.89 (17)	O3—N4—C9	117.10 (17)
C6—C7—N5	119.01 (18)	O5—N5—O6	124.19 (18)
C7—C8—C9	116.48 (17)	O5—N5—C7	117.56 (16)
C7—C8—H8	121.8	O6—N5—C7	118.25 (17)
C9—C8—H8	121.8	C3—O1—Co1	115.13 (12)
C8—C9—C5	123.05 (18)		
			
O2—C3—C4—C6	174.83 (18)	N3^i^—Co1—N1—C2	−81.85 (15)
O1—C3—C4—C6	−3.9 (3)	N2^ii^—Co1—N1—C2	159.19 (14)
O2—C3—C4—C5	−2.1 (3)	N1—C2—N2—N3	0.6 (2)
O1—C3—C4—C5	179.20 (17)	N1—C2—N2—Co1^iii^	−179.28 (12)
C6—C4—C5—C9	0.6 (3)	N1—C1—N3—N2	0.1 (2)
C3—C4—C5—C9	177.53 (16)	N1—C1—N3—Co1^iv^	−178.75 (11)
C5—C4—C6—C7	−0.4 (3)	C2—N2—N3—C1	−0.41 (18)
C3—C4—C6—C7	−177.31 (17)	Co1^iii^—N2—N3—C1	179.45 (12)
C4—C6—C7—C8	−0.1 (3)	C2—N2—N3—Co1^iv^	178.44 (12)
C4—C6—C7—N5	178.47 (17)	Co1^iii^—N2—N3—Co1^iv^	−1.7 (2)
C6—C7—C8—C9	0.4 (3)	C8—C9—N4—O4	−175.39 (17)
N5—C7—C8—C9	−178.19 (16)	C5—C9—N4—O4	1.3 (3)
C7—C8—C9—C5	−0.2 (3)	C8—C9—N4—O3	4.7 (3)
C7—C8—C9—N4	176.34 (17)	C5—C9—N4—O3	−178.66 (18)
C4—C5—C9—C8	−0.3 (3)	C8—C7—N5—O5	174.23 (18)
C4—C5—C9—N4	−176.77 (17)	C6—C7—N5—O5	−4.4 (3)
N3—C1—N1—C2	0.2 (2)	C8—C7—N5—O6	−5.8 (3)
N3—C1—N1—Co1	175.77 (12)	C6—C7—N5—O6	175.59 (17)
N2—C2—N1—C1	−0.5 (2)	O2—C3—O1—Co1	−6.3 (2)
N2—C2—N1—Co1	−176.45 (12)	C4—C3—O1—Co1	172.33 (11)
O1—Co1—N1—C1	−131.24 (16)	N3^i^—Co1—O1—C3	48.42 (14)
N3^i^—Co1—N1—C1	103.46 (16)	N1—Co1—O1—C3	−68.51 (13)
N2^ii^—Co1—N1—C1	−15.50 (18)	N2^ii^—Co1—O1—C3	167.19 (12)
O1—Co1—N1—C2	43.46 (16)		

Symmetry codes: (i) −*x*, *y*−1/2, −*z*+1/2; (ii) *x*, −*y*+1/2, *z*+1/2; (iii) *x*, −*y*+1/2, *z*−1/2; (iv) −*x*, *y*+1/2, −*z*+1/2.
